# *Agrobacterium*-Mediated Transient Gene Expression and Silencing: A Rapid Tool for Functional Gene Assay in Potato

**DOI:** 10.1371/journal.pone.0005812

**Published:** 2009-06-05

**Authors:** Pudota B. Bhaskar, Muthusubramanian Venkateshwaran, Lei Wu, Jean-Michel Ané, Jiming Jiang

**Affiliations:** 1 Department of Horticulture, University of Wisconsin-Madison, Madison, Wisconsin, United States of America; 2 Department of Agronomy, University of Wisconsin-Madison, Madison, Wisconsin, United States of America; Cairo University, Egypt

## Abstract

Potato is the third most important food crop worldwide. However, genetic and genomic research of potato has lagged behind other major crops due to the autopolyploidy and highly heterozygous nature associated with the potato genome. Reliable and technically undemanding techniques are not available for functional gene assays in potato. Here we report the development of a transient gene expression and silencing system in potato. Gene expression or RNAi-based gene silencing constructs were delivered into potato leaf cells using *Agrobacterium*-mediated infiltration. Agroinfiltration of various gene constructs consistently resulted in potato cell transformation and spread of the transgenic cells around infiltration zones. The efficiency of agroinfiltration was affected by potato genotypes, concentration of *Agrobacterium*, and plant growth conditions. We demonstrated that the agroinfiltration-based transient gene expression can be used to detect potato proteins in sub-cellular compartments in living cells. We established a double agroinfiltration procedure that allows to test whether a specific gene is associated with potato late blight resistance pathway mediated by the resistance gene *RB*. This procedure provides a powerful approach for high throughput functional assay for a large number of candidate genes in potato late blight resistance.

## Introduction

Potato (*Solanum tuberosum*) is the third most important food crop in the world, next only to rice and wheat. However, genetic and genomic research of potato has lagged behind most major crops. Functional discovery of genes in potato is still a lengthy process and is often hampered by the complex characteristics associated with the potato genome, including autotetraploidy, self-incompatibility, and high heterozygosity. Although several gene discovery tools have been used in potato research, including transposon-based insertional mutagenesis [1], [2], gene activation-tagging [3], and map-based cloning [4], [5], applications of these techniques were time-consuming, resource-intensive, and technically challenging. RNA interference (RNAi)-based potato gene silencing has recently been reported by several laboratories [6], [7], [8], [9], [10], [11], [12], [13]. However, the RNAi technique relies on the traditional transformation procedure and is a low throughput methodology. It takes on average six months to develop a transgenic potato line using RNAi constructs. Therefore, this technique can only be used to target a limited number of potato genes.

Transient gene assays are convenient alternatives to stable transformation because such techniques allow a rapid analysis of gene function. Early successful transient gene assay in potato was demonstrated using a microprojectile bombardment-based appeoach [14]. Virus-induced gene silencing (VIGS) has been successfully used in several plant species, including potato [15], [16]. However, VIGS has not yet been proven to be a straightforward technique that can be readily adapted in different potato laboratories. As a similar approach to VIGS, transient gene expression was also be accomplished by infection of an *Agrobacterium tumefaciens* strain carrying a potato virus X (PVX)-based binary vector [17]. This approach was successfully used in high-throughput screening for specific recognition of INF elicitins of *Phytophthora infestans* in different *Solanum* species [17]. Leaf infiltration of *Agrobacterium* is another popular method for transient gene functional assay. The agroinfiltration has been best used in *Nicotiana benthamiana* [18], although it has also been successfully applied to several other plant species, including *Arabidopsis thaliana* [19], tobacco [20], [21], tomato [22], lettuce [19], [23], and grapevine [24], [25]. To our knowledge, *Agrobacterium*-mediated infiltration for rapid functional gene assays without involving a viral based system has not been reported in potato.

An international Potato Genome Sequencing Consortium (PGSC) has been established (http://potatogenome.net) and is expected to fully sequence the 850 Mb potato genome by the end of 2010. This soon available genome sequence will dramatically change the genetic and genomic research of potato. One of the most urgently needed tools is a reliable, efficient, and high throughput technique for discovery and characterization of individual potato genes. We have developed an *Agrobacterium*-mediated infiltration procedure in potato. Gene expression or RNAi-based gene silencing constructs can be delivered into potato leaf cells using agroinfiltration. We demonstrated that the agroinfiltration technique can be used as a rapid gene assay tool to localize protein expression in sub-cellular compartments and to determine the role of candidate genes in *R*-gene mediated potato late blight resistance.

## Results and Discussion

### I. Transient gene expression mediated by agroinfiltration

#### Infiltration and plant material optimization

**Figure 1:**
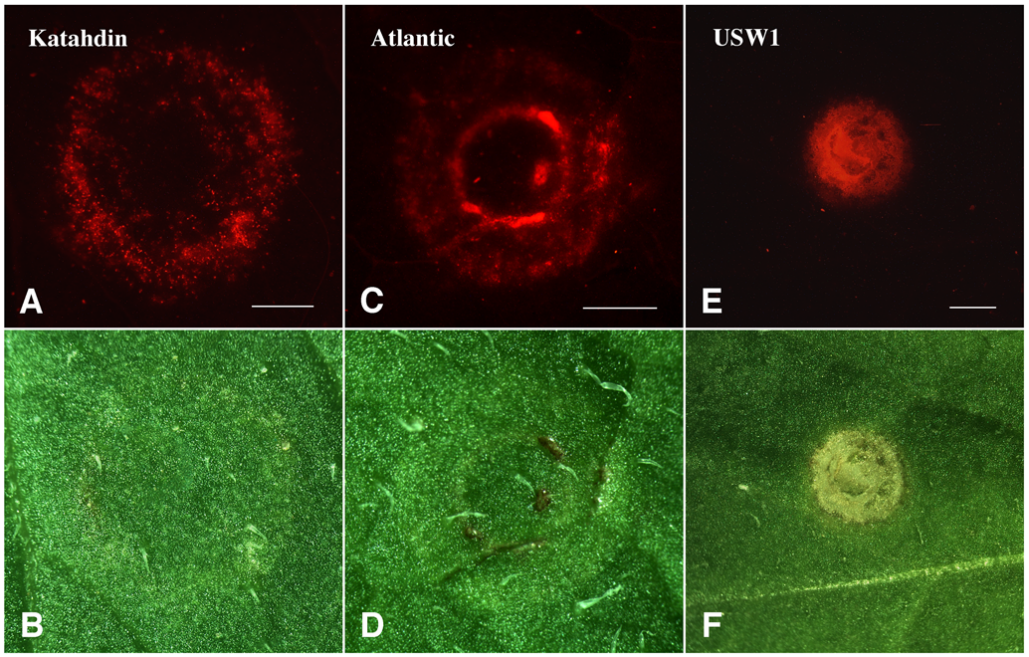
Red fluorescence derived from *Ds*RED1 six days after agroinfiltration into potato leaves. (A) Red fluorescence from a single infiltration site on Katahdin. (B) The same infiltration site as (A) under bright field. (C) Red fluorescence from a single infiltration site on Atlantic. (D) The same infiltration site as (C) under bright field. (E) Red fluorescence from a single infiltration site on USW1. No transgenic cells were detected on this image. The strong red fluorescence signals in this infiltration site were derived from autofluorescence associated with the necrotic tissue. (F) The same infiltration site as (E) under bright field. All bars are 10 mm.

The efficiency and versatility of the agroinfiltration technique in *N. benthamiana* prompted us to test the possibility to adapt a similar approach in potato. Our initial experiments using previously established protocols in *N. benthamiana* [21], [26] only resulted in limited success with a low transformation efficiency. To optimize the procedure in potato, Katahdin, a cultivar highly amenable to whole plant transformation, was used to infiltrate the leaves at various stages of growth. A potato RAR1:: GFP (Required for *Mla12* Resistance, Green Fluorescent Protein) construct, which also contained the *Ds*RED1 (Red Fluorescent Protein) reporter, was used to optimize all the infiltration conditions. The presence and spread of transgenic cells around the infiltration zones were identified based on red fluorescence under an epifluorescence microscope (Fig. 1). In contrast, no *Ds*RED1 fluorescence was observed from un-infiltrated potato leaves.

We tested the potato leaves at various growth ages and found that the infiltration was consistently most efficient when using terminal leaflets from 5–6 week-old potato plants. We noticed that the leaflets from middle or lower positioned leaves with less pubescence were easier to infiltrate. The efficiency of infiltration became significantly lower when leaves from 3–4 week old plants were used in the experiments. We then investigated whether the concentration of the *Agrobacterium* cultures had any effect on the outcome of infiltration. Cultures resuspended to OD_600_ = 0.2–0.5 resulted in the best transient gene expression activity. In order to suppress the silencing of the transgene, we co-infiltrated the silencing suppressor P19 [27] together with the transgene. However, the introduction of P19 appeared to have no effect on the expression of transgenes. We used two different *Agrobacterium* strains, GV3101 and LBA4404, for infiltration. GV3101 showed a higher efficiency than LBA4404 in the experiments, which confirms the high efficiency of GV3101 reported in *N. benthamiana* [21], [26].

#### Evaluation of transient expression using different potato genotypes

We infiltrated leaves from potato cultivars Katahdin, Atlantic, Megachip, USW1, and a wild diploid species *Solanum bulbocastanum* in order to investigate the influence of potato genotypes on the efficiency of transient expression. We observed a considerable variation for the intensity of the *Ds*RED1 fluorescence among the genotypes. Katahdin showed the highest transformation efficiency. We consistently observed a large number of transgenic cells away from the infiltration zone in Katahdin leaves (Fig. 1A). Katahdin was followed by Atlantic (Fig. 1C) and Megachip where the transgenic cells were mostly observed in close proximity to the infiltration zone. Transformation events were not observed in leaves from USW1 (Fig. 1E) and *S. bulbocastanum* (data not shown).

Several previous studies reported that potato genotypes, both different potato cultivars and different *Solanum* species, can affect the success of the VIGS technique [15] and agro-infection assays [17], [28]. Similarly, studies in grapevine also showed an effect of genotype for success of infiltration assays [24]. The high infiltration efficiency with Katahdin leaves in our study is in accordance with the high efficiency of this cultivar in whole plant transformation. We thus used this Katahdin and *Agrobacterium* strain GV3101 for all further studies. The transient functional assay using agroinfection of PVX-based constructs will depend on the susceptibility of various potato cultivars to PVX. Thus, the agroinfiltration method offers an alternative strategy to the viral based systems.

#### Sub-cellular localization of various potato proteins

**Figure 2:**
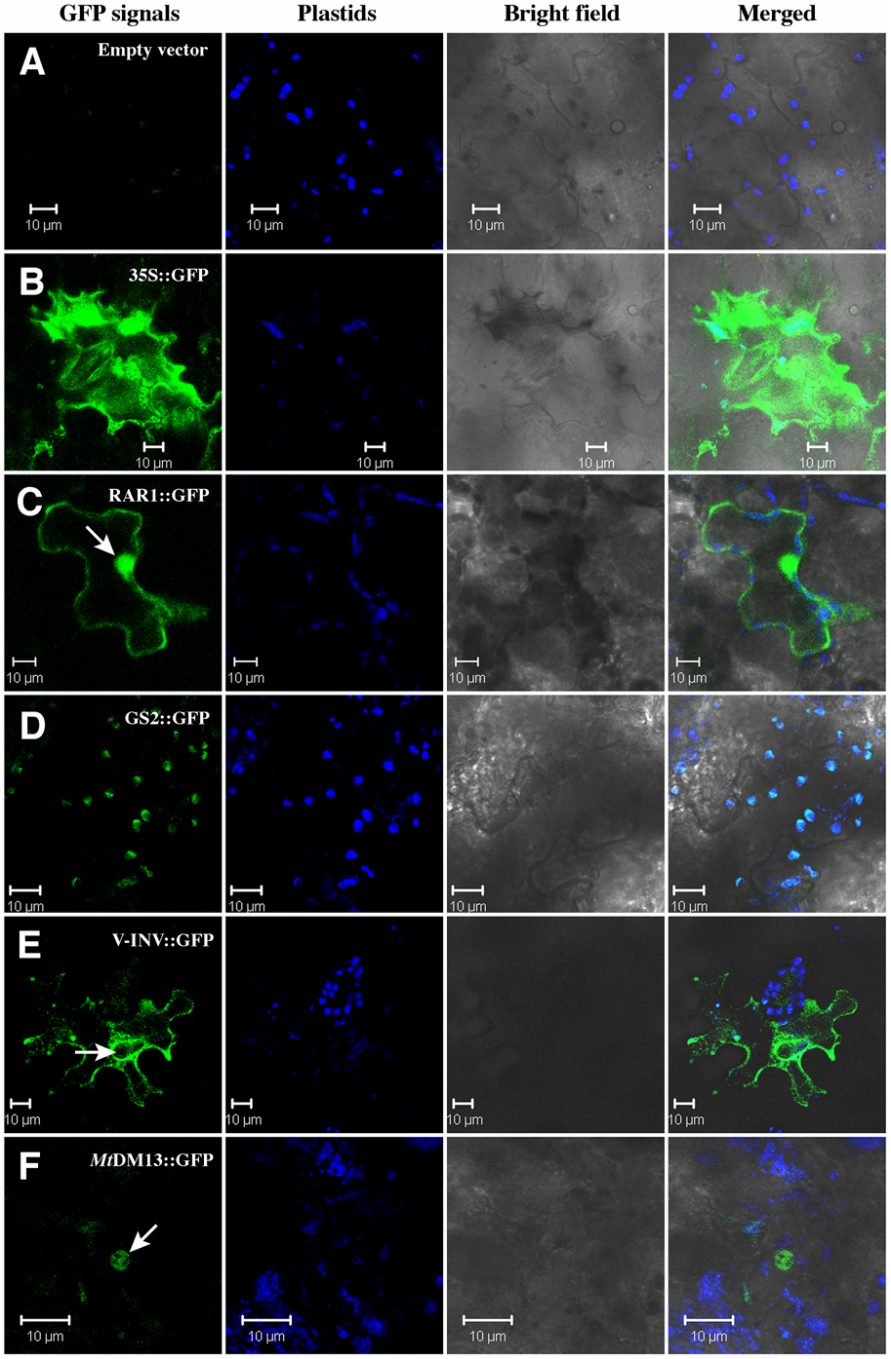
Laser-scanning confocal micrographs showing GFP fluorescence from agroinfiltrated leaf cells. Katahdin leaves were agroinfiltrated with (A) pK7FWG2 empty vector; (B) 35S::GFP; (C) *St*RAR1::GFP; (D) *St*GS2::GFP; (E) *St*V-INV::GFP; and (F) *Mt*DMI3::GFP. The background fluorescence derived from plastids is in blue color. All the scale bars represent 10 µm. Arrows point to the nucleus in the cells.

One of the main applications of the transient gene assay tools is to monitor the localization of proteins to sub-cellular compartments in living cells. To test such a function of our agroinfiltration technique we developed several GFP-tagged potato gene constructs. Katahdin leaves showed only minimum autofluorescence under a GFP filter five and seven days after agroinfiltration with the pK7FWG2 empty vector (Fig. 2A). In contrast, a vector expressing GFP under the 35S promoter (35S::GFP) showed strong GFP fluorescence signals throughout the cellular compartments (Fig. 2B).

Next we examined a few potato genes for which the protein locations within specific sub-cellular compartments have been either known or can be predicted. Full-length coding sequences (CDS) of the target genes were amplified and cloned into the binary vector pK7FWG2. The C-terminal of the CDS was fused with the GFP gene. The constructs were then agroinfiltrated into potato leaves. We first determined the sub-cellular localization of the *St*RAR1::GFP fusion proteins. The GFP-tagged potato RAR1 proteins were ubiquitously localized in the cytoplasm as well as in the nucleus (Fig. 2C). The GFP signals present in the nucleus were much stronger than those in the cytoplasm. These results were in agreement with the cellular localization of rice *Os*RAR1::GFP [29]. *St*GS2::GFP (Potato Glutathione Synthetase-2, GS2), an enzyme involved in the synthesis of glutathione in plants, was found to be predominantly localized within plastids with no unambiguous signals in other cellular compartments (Fig. 2D). The GS2 protein was previously showed to be exclusively targeted to plastids in *A. thaliana*, *Brassica juncea* [30] and *Medicago truncatula* [31]. Our results were in confirmation with these previous studies.

We then tested cellular localization of the potato vacuolar invertase (*St*V-INV) protein, which was not studied previously. The *St*V-INV::GFP protein was localized in cytoplasm, including endoplasmic reticulum (ER) and vacuoles (Fig. 2E). Conclusive GFP signals were not detected in the nucleus. Interestingly, the protein appeared also to be localized around the nucleus and in the cytoplasm. However, this signal pattern may be due to the strong GFP signals from ER surrounding the nuclear envelope. These results agreed with the prediction of protein localization site analysis (PSORT, www.psort.org) that the 1,920 bp CDS of potato *V-Inv* (639 aa of V-INV protein) to be primarily localized in ER, Golgi bodies, endosomes (peroxisomes), and membrane system within the cells.

The *M. truncatula* DMI3 protein, which is required for the initiation of legume nodulation, has previously been shown to be localized in the nucleus [32], [33]. We expressed the *Mt*DMI3::GFP fusion protein into the potato cells. The GFP signals were predominantly localized in the nucleus in most of the transgenic cells (Fig. 2F). These results show that the agroinfiltration-based technique can be used to study both native and heterologous proteins in potato.

### II. RNAi-based transient gene silencing mediated by agroinfiltration

#### Optimization of the transient silencing procedure

We initially used the established optimal agroinfiltration procedure described above for RNAi-based transient gene silencing experiments. However, we observed that young and fully expanded leaves from 3–5 week-old plants were the best plant materials for transient gene silencing. Maximum silencing levels were obtained when *Agrobacterium* inoculum was diluted to an OD_600_ value of 0.3–0.7. A low concentration (OD_600_<0.1) of bacterial suspension resulted in poor transformation thus leading to lower silencing levels, while high concentration (OD_600_>1.0) occasionally produced necrotic spots around the infiltration zones.

**Figure 3:**
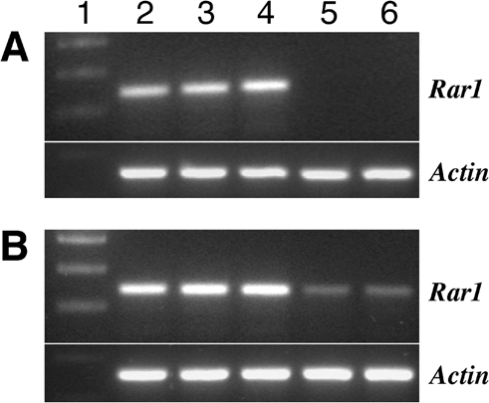
RT-PCR analysis of transient silencing of the potato *Rar1* gene in two independent potato leaves (A and B). Leaf samples around the infiltrated spots were collected at days 1, 2, 5 and 6 dpi. Lane 1: 100 bp DNA ladder marker; Lane 2: leaf sample from un-infiltrated control; Lane 3: leaf from infiltrated site 1 dpi; Lane 4: leaf from infiltrated site 2 dpi; Lane 5: leaf from infiltrated site 5 dpi; Lane 6: leaf from infiltrated site 6 dpi. *Actin* was amplified as a control for the amount of template. The amplified *Rar1* and *Actin* transcripts are 339 bp and 360 bp, respectively.

We used a previously developed *Rar1*-*RNAi* construct [9] in our initial transient gene silencing experiments. This construct was agroinfiltrated into Katahdin leaves. Semi-quantitative RT-PCR was used to confirm the suppression of the potato *Rar1* gene in the leaf tissues around the infiltration zone. The *Rar1* transcript was significantly reduced compared with the controls (Fig. 3). Reduction of the *Rar1* transcript was generally observed 5 and 6 days post infiltration (dpi) and persisted until 8 dpi. However, partial reduction of the *Rar1* transcripts started as early as 3–4 dpi in 20–30% of the leaves analyzed. The reduction of the *Rar1* transcript compared to the control leaves was as much as 90–99% in different experiments (Fig. 3). Our results are in accordance with the previous finding in *N. benthamiana* that the production of siRNAs for the target gene in the infiltrated zones started as early as 2 days post infiltration and reached a peak abundance by day 5 [34].

#### Rapid late blight resistance assay using RNAi-based transient silencing

Agroinfiltration assay was successfully used for screening candidate signaling components required for the activation of *R*-gene mediated disease resistance in *N. benthamiana* and tomato [26], [35]. We intended to develop a similar technique to screen the candidate genes required for the late blight resistance mediated by the *RB* gene. Gene *RB* confers a broad-spectrum resistance against the late blight pathogen *Phytophthora infestans* [5] and recognizes the *P. infestans* effector, IpiO1 [28]. In our procedure an *RNAi* construct developed against a candidate gene was first introduced into a *RB*-containing potato plant by agroinfiltration, which will silence the target gene. Four days after agroinfiltration, the second construct containing the *IpiO1* gene was agroinfiltrated at the same site. This double infiltration would result in a hypersensitive response (HR) phenotype if the candidate gene is not involved in the *RB*-mediated resistance, because the silencing of this candidate gene will not affect the resistance. However, no HR would be observed if the candidate gene is involved in *RB*-mediated resistance.

**Figure 4:**
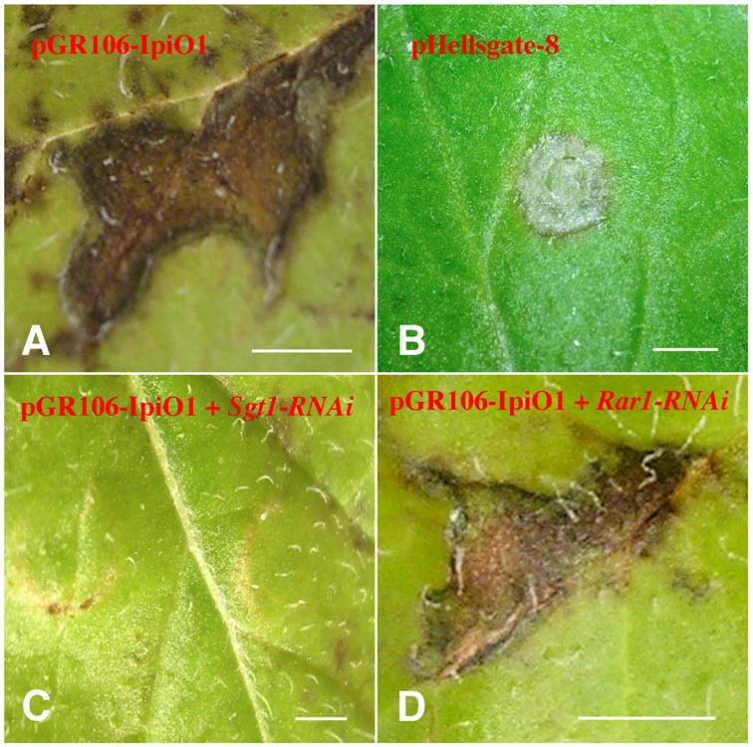
A double agroinfiltration procedure to test candidate genes associated with potato late blight resistance mediated by the *RB* gene. All the pictures were taken at 10 days post infiltration and bars represent 2 cm. (A) Infiltration with *Agrobacterium* carrying pGR106-IpiO1 and HR response was observed around the infiltrated site. (B) Infiltration with *Agrobacterium* containing pHellsgate-8 silencing construct. (C) Double agroinfiltration with *Agrobacterium* carrying *Sgt1-RNAi* construct followed with pGR106-IpiO1. No HR was observed around the infiltrated site. (D) Double agroinfiltration with *Agrobacterium* carrying *Rar1-RNAi* construct followed with pGR106-IpiO1. HR response was observed around the infiltrated site.

A transgenic Katahdin line SP925, which contains the *RB* gene, was used in the double agroinfiltration experiments. The *Agrobacterium* strain GV3101 produced few or no necrotic spots on *RB*-Katahdin leaves 7–12 dpi (Fig. 4). In contrast, infiltration with effector IpiO1 in the same plant resulted in a confluent necrosis response starting at 3–5 dpi (Fig. 4). Infiltrations with silencing construct alone or mock infiltration did not produce any background effect on potato leaves observed until 10 dpi, although few necrotic spots emerged 10 dpi even in the control experiments, which may be caused by the natural senescence of the tissues around the site of infiltration.

The *Rar1* and *Sgt1* genes have been extensively studied for their roles in the regulation of disease resistance genes [36]. We have previously demonstrated that SGT1, but not RAR1, is essential for the *RB*-mediated late blight resistance in potato [9]. We performed double agroinfiltration using *Rar1*-*RNAi* and *Sgt1*-*RNAi* constructs together with the *IpiO1* gene construct. Eight days after the first infiltration, a clear HR response was observed around the infiltrated sites on potato leaves double infiltrated with *Rar1*-*RNAi* constructs and IpiO1 (Fig. 4). In contrast, no HR was observed around the infiltrated sites of *Sgt1*-*RNAi* and IpiO1 (Fig. 4). These results showed that *RB* activation by IpiO1 depends on SGT1 but not RAR1, which is consistent with our whole plant stable transformation results [9].

Late blight is the most devastating potato disease worldwide and is also the most extensively studied potato disease [37]. Several late blight resistance genes, including both race-specific and race-non-specific genes, have been cloned in recent years [4], [5], [38], [39], [40], [41]. However, very limited effort so far has been devoted to understand the resistance pathways mediated by any of these genes. This is at least partially due to the lack of tools in potato for rapid analysis of candidate genes associated with resistance or signaling pathway. The double agroinfiltration technique developed in this study will provide a powerful tool to fill this need in the future.

### Conclusions

The soon available potato genome sequence will dramatically accelerate our pace to identify agronomically important potato genes. The power of comparative genomics will also allow us to discover potato genes based on the information from other extensively studied model plant species. Thus, a rapid and simple functional gene assay tool is urgently needed for potato genetics and molecular biology research. We demonstrate that *Agrobacterium*-mediated infiltration, which has been an effective gene delivering technique in several plant species, can be adapted in potato. Katahdin, a potato cultivar that is highly amenable for *Agrobacterium*-mediated whole plant transformation, showed the highest efficiency for agroinfiltration in our study. However, it will be possible to identify potato cultivars (genotypes) that have greater efficiency for agroinfiltration than Katahdin. Agroinfiltration of GFP-based gene expression constructs into potato leaf cells is a simple and highly efficient approach to examine protein expression in sub-cellular compartments. We also demonstrate that double agroinfiltration of RNAi-based silencing construct and a late blight pathogen effector can be used for screening candidate genes involved in late blight resistance pathway mediated by the corresponding resistance gene. This double agroinfiltration approach is simple and fast compared to the traditional approach consisting of stable transformation followed by disease resistance evaluation [9]. It can be readily adapted to dissect the resistance pathways mediated by a wide range of potato *R* genes in the future.

## Materials and Methods

### Plant materials

Potato cultivars Katahdin, Atlantic, Megachip, USW1 (a haploid clone derived from Katahdin), and a diploid wild potato species *S. bulbocastanum* were used for transient gene expression experiments. Katahdin and a *RB*-transgenic Katahdin line, SP925 [42] were used for RNAi-based transient silencing assays. All the plants were maintained in the greenhouse facility of the University of Wisconsin-Madison. Growth conditions with 70% humidity, 16-h day/8-h night regime, 19°C/15°C, 500 µmol m^−2^ s^−1^ light were applied.

### Plasmid construction and detection

The constructs used for transient gene expression and localization studies were derived from binary vector pK7FWG2, which allows C-terminal fusion of protein of choice with GFP [43]. These vectors were obtained from the Ghent University, Belgium. A modified pK7FWG2-R vector containing an additional *DsRED1* marker driven by *AtUBQ10* [32] was used to evaluate the transformation efficiency in different potato genotypes. The 675-bp CDS of the potato *Rar1* gene (TIGR potato EST TC121848; http://compbio.dfci.harvard.edu/tgi/cgi-bin/tgi/gireport.pl?gudb=potato) was amplified without its stop codon using the primers 5′-CACC ATG GAG AGA CTT CGA TGT CAG AGG-3′ (forward) and5′-GGA CAC TGG GTC AGC GTT GTG C-3′ (reverse). The sequence verified PCR products were cloned into the pENTR/D-TOPO vector using the pENTR Directional TOPO Cloning kit (Invitrogen, Carlsbad, California). The products were then transferred to the pK7FWG2 vector by LR recombination reaction (Invitrogen), resulting in 35S::*St*RAR1::GFP fusion. The same products were also transferred to pK7FWG2-R modified binary vector. A 1642 bp CDS of the potato Glutathione Synthetase gene (GenBank Accession, AF017984) without a stop codon was amplified using the primers 5′-CACC ATG GGC AGC GGC TGT TCT TCT CCA-3′ (forward) and 5′-AAC CAA GTA TAT ACT GTC CAA AA-3′ (reverse), transferred into binary vector pK7FWG2 to obtain 35S::*St*GS2::GFP fusion. Similarly, a 2,030-bp CDS of the potato Vacuolar Invertase gene (TIGR Accession ID, TC132799) was amplified without a stop codon using the primers 5′-CACC ATG GCC ACC CAG TAC CAT TCC AGT-3′ (forward) and 5′-CAA GTC TTG CAA GGG GAA GGA TCG-3′ (reverse), transferred into binary vector pK7FWG2 to obtain 35S::*St*V-INV::GFP fusion. A *Mt*DMI3::GFP fusion construct was described previously [32], [33].

All PCR amplifications were performed using Platinum *Taq* DNA polymerase (Invitrogen) and leaf cDNA from Katahdin was used in all amplifications. Electro-competent cells of *A. tumefaciens* strain GV3101 were prepared and transformed as described previously [44]. *Agrobacterium* was also transformed with empty vectors pK7FWG2 and pK7FWG2-R to generate control plasmids. Transformation was achieved by using 2 µl of purified construct DNA per 30 µl of *Agrobacterium* competent cells (GV3101) shocked at 2.2 kV voltage and 25 µF capacitance using an electroporator. After two hours of incubation at 28°C, the cells were plated on selective media (spectinomycin, gentamycin, or rifamycin) and grown for 1–3 days at 28°C.

For transient gene silencing assay, *Agrobacterium* strain LBA4404 harboring either *Rar1-RNAi* or *Sgt1-RNAi* construct [9] was used as previously described for transient silencing experiments. An empty vector construct was obtained by transforming *Agrobacterium* strain LBA4404 with the pHellsgate-8 silencing plasmid [45]. The same electroporation method was followed described above for obtaining fusions. *Agrobacterium* strain, GV3101 harboring pGR106-Ipio1 (effector) was described previously [28].

### Agroinfiltration and imaging

A single colony of recombinant *Agrobacterium* strain of GV3101 was cultured in 5 ml of LB culture containing antibiotics spectinomycin (50 mg/ml) and rifamycin (25 mg/ml) and grown overnight (28°C at 225 rpm). A large LB media suspension was then inoculated with the overnight culture and grown at 28°C to an OD_600_ of ∼1.0. cells. The cells were harvested by centrifugation at 5500 rpm for 2 min and resuspended in 1 ml of infiltration buffer (10 mM MgCl_2_ and 100 µM acetosyringone). This step was repeated at least once and the concentration of bacterial suspension was measured by spectroscopy (OD_600_) and adjusted to a final desired concentration with the infiltration buffer and left at room temperature for 1–2 h. An OD_600_ of 0.2–0.5 was adjusted for transient expression studies. The bacterial suspension was taken in a syringe and infiltrated through the abaxial surface of the leaf. Before infiltration, a small incision was made at the site of infiltration using a sterile toothpick to enhance the efficiency of infiltration. Five to six days after infiltration, the localization of GFP-fused proteins was observed using Zeiss LSM 510 meta inverted confocal laser microscope. A 1×1 cm^2^ leaf section was taken 3–5 cm away from the infiltration zone for GFP signal analysis. The *Ds*RED fluorescence was observed by separating infiltrated leaf from the plant, 6 days post infiltration, and observed under Leica MZ16-F epifluorescence microscope under a *Ds*RED filter set.

The same procedure described above for transient expression assays with only minor modifications. *A. tumefaciens* cells were harvested by centrifuging at 5500 rpm for 20 min and resuspended in 5 ml infiltration buffer containing 10 mM MES (pH 5.7), 10 mM MgCl_2_ and 100 µM acetosyringone. An OD_600_ of 0.3–0.7 was adjusted for transient silencing studies and suspensions were left at room temperature for 3 hrs. For double agroinfiltration experiments, leaves from a *RB*-containing transgenic Katahdin line SP925 were infiltrated with *A. tumefaciens* strain carrying either *Rar1-RNAi* or *Sgt1-RNAi* construct [9] with an OD_600_ of 0.3–0.7. Four days later, the same infiltration sites were challenged with an *A. tumefaciens* strain carrying the pGR106-IpiO1 plasmid. A similar OD_600_ of 0.3–0.7 was adjusted for these infiltrations.

### RNA isolation and RT-PCR analysis

For collecting leaf samples for RNA isolation and RT-PCR analysis, a 1×1 cm^2^ incision was made using a razor blade surrounding the infiltration zones (1–3 cm away from the infiltration zones). The collected samples were frozen immediately in liquid nitrogen and total RNA was extracted from leaf tissue samples collected at various time points using the RNeasy Plant Mini Kit (Qiagen, Valencia, California). All the samples were treated with TURBO DNA-*free* (Ambion, Austin, Texas) to remove DNA contamination. First strand cDNA was synthesized using 1.5 µg of total RNA, oligo d(T) primer and superscript reverse transcriptase (Invitrogen). Semi-quantitative RT-PCR was performed as described in Bhaskar et al. (2008). For RT-PCR, primers that anneal outside the region targeted for silencing were used to ensure that the gene of interest was silenced. Primers 5′-TTG CAA CGC TAC CTT CAC TG-3′ (forward) and 5′- GGA ATT GCT TCT CTG GGT TG-3′ (reverse) were used to amplify a 339 bp of *Rar1* cDNA. Primers 5′-GAT GGC AGA CGG AGA GGA TA-3′ (forward) and 5′- CAC GAT TAG CCT TGG GGT TA-3′ (reverse) were used to amplify a 360 bp of *Actin* cDNA.
